# One step forward: contrasting the effects of Toe clipping and PIT tagging on frog survival and recapture probability

**DOI:** 10.1002/ece3.1047

**Published:** 2014-03-24

**Authors:** Murilo Guimarães, Décio T Corrêa, Sérgio S Filho, Thiago A L Oliveira, Paul F Doherty, Ricardo J Sawaya

**Affiliations:** 1 Programa de Pós-Graduação em Ecologia, Departamento de Biologia Animal, Instituto de Biologia, Universidade Estadual de CampinasCP 6109, Campinas, SP, cep 13083-970, Brazil; 2 Ecology, Evolution, and Behavior Graduate Program, Department of Integrative Biology, The University of Texas at Austin1 University Station #C0930, Austin, Texas, 78712; 3 Programa de Pós-Graduação em Biologia Animal, Departamento de Zoologia e Botânica, Universidade Estadual PaulistaSão José do Rio Preto, SP, Cep 15054-000, Brazil; 4 Fish, Wildlife and Conservation Biology Department, Colorado State UniversityPO 80523, Fort Collins, Colorado; 5 Departamento de Ciências Biológicas, Universidade Federal de São PauloDiadema, SP, Cep 09972-270, Brazil

**Keywords:** Amphibians, detection probability, Hylidae, mark–recapture, multimodel inference, return rate

## Abstract

Amphibians have been declining worldwide and the comprehension of the threats that they face could be improved by using mark–recapture models to estimate vital rates of natural populations. Recently, the consequences of marking amphibians have been under discussion and the effects of toe clipping on survival are debatable, although it is still the most common technique for individually identifying amphibians. The passive integrated transponder (PIT tag) is an alternative technique, but comparisons among marking techniques in free-ranging populations are still lacking. We compared these two marking techniques using mark–recapture models to estimate apparent survival and recapture probability of a neotropical population of the blacksmith tree frog, *Hypsiboas faber*. We tested the effects of marking technique and number of toe pads removed while controlling for sex. Survival was similar among groups, although slightly decreased from individuals with one toe pad removed, to individuals with two and three toe pads removed, and finally to PIT-tagged individuals. No sex differences were detected. Recapture probability slightly increased with the number of toe pads removed and was the lowest for PIT-tagged individuals. Sex was an important predictor for recapture probability, with males being nearly five times more likely to be recaptured. Potential negative effects of both techniques may include reduced locomotion and high stress levels. We recommend the use of covariates in models to better understand the effects of marking techniques on frogs. Accounting for the effect of the technique on the results should be considered, because most techniques may reduce survival. Based on our results, but also on logistical and cost issues associated with PIT tagging, we suggest the use of toe clipping with anurans like the blacksmith tree frog.

## Introduction

With the current state of amphibian declines (Stuart et al. 2004), quantitative links between vital rates and explanatory covariates are fundamental to understand the dynamics of and threats to populations (Biek et al. 2002). The results obtained by marking individuals provide accurate information on population trends and demographic estimates (Manly et al. 2005), especially when population dynamics are poorly understood, as in the Neotropics (Hiert et al. 2012).

Although field biologists strive to apply the least harmful marking technique to their study species, most techniques remain at least somewhat invasive and may affect individual behavior and survival (Lemckert 1996; Bloch and Irschick 2004; Ferner 2007; Schmidt and Schwarzkopf 2010). Among the different techniques used to mark anurans (Donnelly et al. 1994), the most common is toe clipping (Bogert 1947), which consists of removing different combinations of digits to give individuals unique marks.

Nevertheless, the scientific community has divergent opinions regarding the impacts of marking individuals, especially via toe clipping (May 2004; Funk et al. 2005). Besides that, environmental agencies and common sense from different countries have also expressed concerns about the efficacy of such a potential unappealing technique for studying frogs and other vertebrates (Ferner 2007). Their opinion derives from several recent papers that have related the number of toes clipped to individual response of amphibians, including low return or survival rates (Parris and McCarthy 2001; McCarthy and Parris 2004; Waddle et al. 2008). In fact, philosophical and legal views deserve attention as methodological efficacy is not the only concern when considering toe clipping (for a review, see Perry et al. 2011). In Brazil, for instance, environmental agencies and nongovernmental organizations claim that toe clipping is a form of mutilation and its use should be prohibited (Corrêa et al. 2013). In general, ethical standard policies on animal welfare state that marking techniques should not cause distress or inflict pain, reducing individual survival. And because of the controversial results to date, Brazil suggested the use of alternative marking techniques, including visible implanted elastomers or photographs of natural marks (Brown 1997; Hoffmann et al. 2008; Campbell et al. 2009; Kenyon et al. 2009), but these methods remain to be contrasted.

The passive integrated transponder (PIT tag) is used worldwide and recommended as an alternative to toe clipping (Donnelly et al. 1994; Gibbons and Andrews 2004; Phillott et al. 2008). It consists of a glass-encapsulated electromagnetic coil with a unique alphanumeric code. The tag is lodged under the skin or in the body cavity of an animal and read by a handheld scanner (Gibbons and Andrews 2004). Because of the possibility to mark a great number of individuals, PIT tags have been used in anurans as an alternative marking technique (Christy 1996; Jehle and Hödl 1998). Nevertheless, negative effects on frog survival have been reported (Scherer et al. 2005), and little is known on the impacts of PIT tags on anurans (e.g., Christy 1996; Brown 1997; Phillott et al. 2008), which may include behavioral and physiological deleterious effects from the injection of the tag. Overall, direct comparisons between different marking techniques in frogs are lacking, which does not allow discussion and the clarification of the effects of marking to advance.

Another issue for studies that have attempted to quantify the effects of marking techniques on frogs is that studies should explicitly consider individual detectability. Past studies looking at toe-clipping effects have used the return rate (e.g., McCarthy and Parris 2004). The return rate assumes that detection probability does not change, which is unrealistic in natural systems due to behavioral heterogeneity (e.g., between sexes) and climatic conditions, such as rainfall, which influences amphibian activity (Duellman and Trueb 1986). Despite the number of studies reporting decreased return rates with increasing number of toes removed, only a few studies have incorporated detection probability (e.g., Waddle et al. 2008; Grafe et al. 2011), which is likely less than one, into survival estimates. If recapture probability differs among groups, but not survival probability, one could conclude through the use of return rates, that toe clipping reduces survival when in fact only recapture probability is reduced.

To further advance the discussion on the topic, we contrasted the effects of toe clipping and PIT tagging on a free-ranging neotropical tree frog population using mark–recapture models, which allowed us to disentangle survival and recapture probabilities (Schmidt 2003; Waddle et al. 2008). We specifically compared survival and recapture probabilities between marking techniques while controlling for sex differences. Our intention is to establish a direct comparison between two of the commonest marking techniques and to provide scientific basis on amphibian conservation biology for field biologists and policy makers.

## Materials and Methods

### Study site and study species

We conducted this study in a 970-m^2^ permanent pond in Estação Ecológica de Jataí (21°33′59.75″ S, 47°43′33.19″ W), a protected area in the state of São Paulo, southeastern Brazil. The reserve is located in a transitional area between the Atlantic Forest and Cerrado biomes, composed of open grassy areas and semi-deciduous forests. Average temperature in the coldest months (June to August) is about 11°C, and about 30°C in the hottest months (December to February). Annual rainfall is about 1500 mm. Precipitation during the rainy season (October to March) typically exceeds 270 mm per month, but does not exceed 27 mm per month during the dry season (April to September).

We sampled an adult population of the blacksmith tree frog *Hypsiboas faber* (Anura, Hylidae, Appendix A), a large tree frog (snout-vent length = 92.3 ± 4.8 mm, *N *= 305; this population) distributed from northern Argentina to eastern Brazil (Martins 1993). As in most amphibian species behavior is sexually divergent, and males *H. faber* occupy a pond and build nests at the beginning of the breeding season, generally from October to March (pers. obs. D.T.C.), then begin to vocalize until a female approaches to inspect the nest (Martins and Haddad 1988).

**Appendix A:**
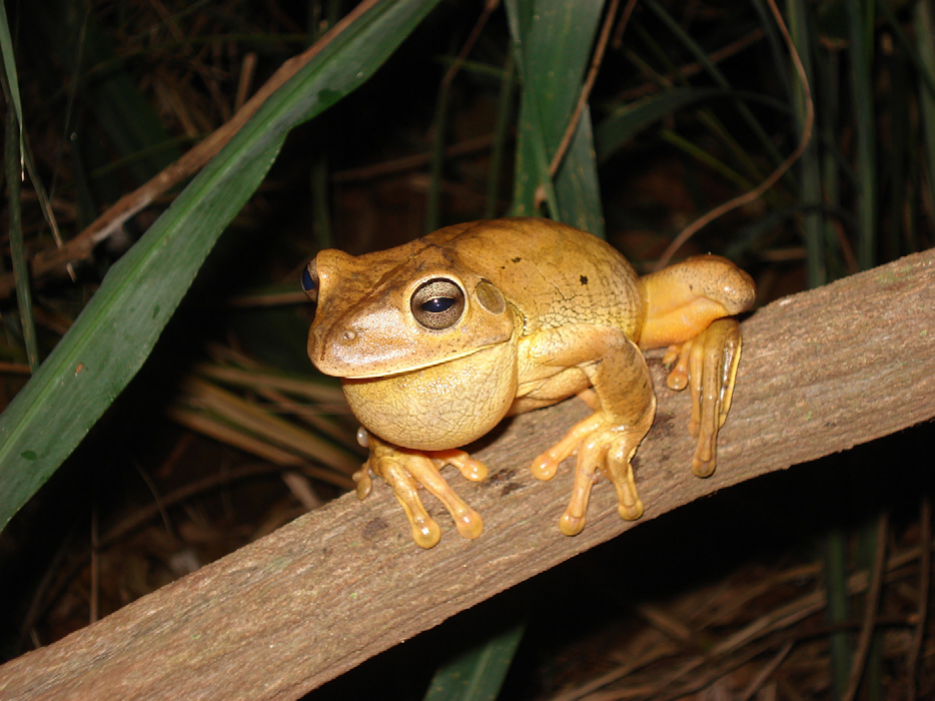
An adult male *Hypsiboas faber*. Photo credit: D. T. Corrêa.

### Data Collection

We collected data during two reproductive seasons, from November to March, in 2010–2011 and 2011–2012. We captured individuals during three nights per month and pooled nights within the same month together, resulting in 10 sampling occasions, five for each breeding season. On each capture occasion, three observers systematically walked around the pond covering from the margins until approximately 150 cm of water depth. Adult individuals were captured by hand based on visual and acoustic cues in all accessible microhabitats.

We determined sex and randomly assigned one type of marking technique to each individual, toe clipping or PIT tagging (IBAMA permit number: 10423-1, COTEC permit number: 010.157/2010). We placed individuals in four different groups: one toe clipped, two toes clipped, three toes clipped, and PIT tags.

For toe-clipping groups, we adapted the marking technique of Waichman (1992), removing only the toe pad (“toe tipping”, *sensu* Phillott et al. 2007), which is enough for individual recognition as tissue regeneration is rarely observed (Lüddecke and Amézquita 1999; Phillott et al. 2007; Grafe et al. 2011). We marked individuals in the toe pad-clipping groups starting with the removal of one toe pad. When all combinations for removing one toe pad were used, we started removing two toe pads in unique combinations, and finally, three toe pad removal combinations, clipping up to two toe pads per limb. This sampling design generates uneven toe pad removal groups over the study period (see Grafe et al. 2011), which we accounted for by including a linear trend model in the parameter estimates (see below).

The PIT-tagging group received a 2.2 mm × 12.2 mm, 0.5 g internal transponder (Animall Tag Company) implanted in a posterior laterally dorsum position, using sterilized needles and followed by the use of glue to help healing. The PIT tag/tree frog mass ratio was 1%. To control for possible effects of the glue, all individuals from the toe pad-clipping groups also received the same amount of glue on the same body region of the PIT tag.

The effects of marking technique and number of toes tipped were assessed in three different ways: (1) by comparing the effects of toe pad clipping against PIT tagging, where we combined groups one, two, and three and compared with group four (hereafter “marking technique effect”); (2) by comparing the survival on individuals with one, two, three toes pad clipped and PIT tagged (hereafter “group effect”); and finally (3) by forcing a linear trend effect only on toe pad-clipping groups (hereafter “linear trend effect”), to test the hypothesis that removal of more toes decreased survival. Also, the inclusion of a linear trend in the recapture probability was used to account for the uneven toe pad removal groups over the study. We used sex as an individual covariate as we expect differences between males and females.

### Statistical analysis

We obtained maximum likelihood parameter estimates using a Cormack–Jolly–Seber (CJS) model (Cormack 1964; Jolly 1965; Seber 1965) in Program MARK version 6.1 (White and Burnham 1999). The CJS model estimates the apparent survival probability (Φ), which is a combination of true survival and site fidelity, and recapture probability (*P*). We used the strategy proposed by Doherty et al. (2010) to run all possible additive combinations of factors (marking techniques and sex), except for combinations that did not make logical sense (e.g., different representations of marking effects not considered a priori). Such a strategy is recommended over stepwise procedures (Doherty et al. 2010), but it may generate a large number of models.

Goodness-of-fit and a variance inflation factor (i.e., median *ĉ*) were assessed using the general model with no individual or temporal covariates (Φ_group_ *P*_group_) to test the mark–recapture assumptions. We selected and ranked models using Akaike Information Criterion (Akaike 1973) adjusted for small sample sizes (AIC*c*, Burnham and Anderson 2002). Survival and recapture probabilities were then model averaged, a weighted average of the model-specific parameter estimates based on Akaike weights, to include uncertainty in model selection (Burnham and Anderson 2002). We then calculated the relative importance of each covariate through the cumulative AIC*c* weights to determine the important covariates for each parameter. Following Barbieri and Berger (2004), we considered covariates with cumulative AIC*c* weight above 0.5 to be important.

## Results

Eighteen individuals (14 males and four females) had one toe pad clipped, 150 (110 males and 40 females) had two toe pads clipped, another 150 individuals (120 males and 30 females) had three toe pads clipped, and 227 individuals (177 males and 50 females) were PIT tagged. We recaptured 117 of 545 individuals at least once. The goodness-of-fit test showed no problem with transient individuals or trap dependence effects, and no extra binomial variation was detected (*ĉ *=* *0.96).

**Figure 1:**
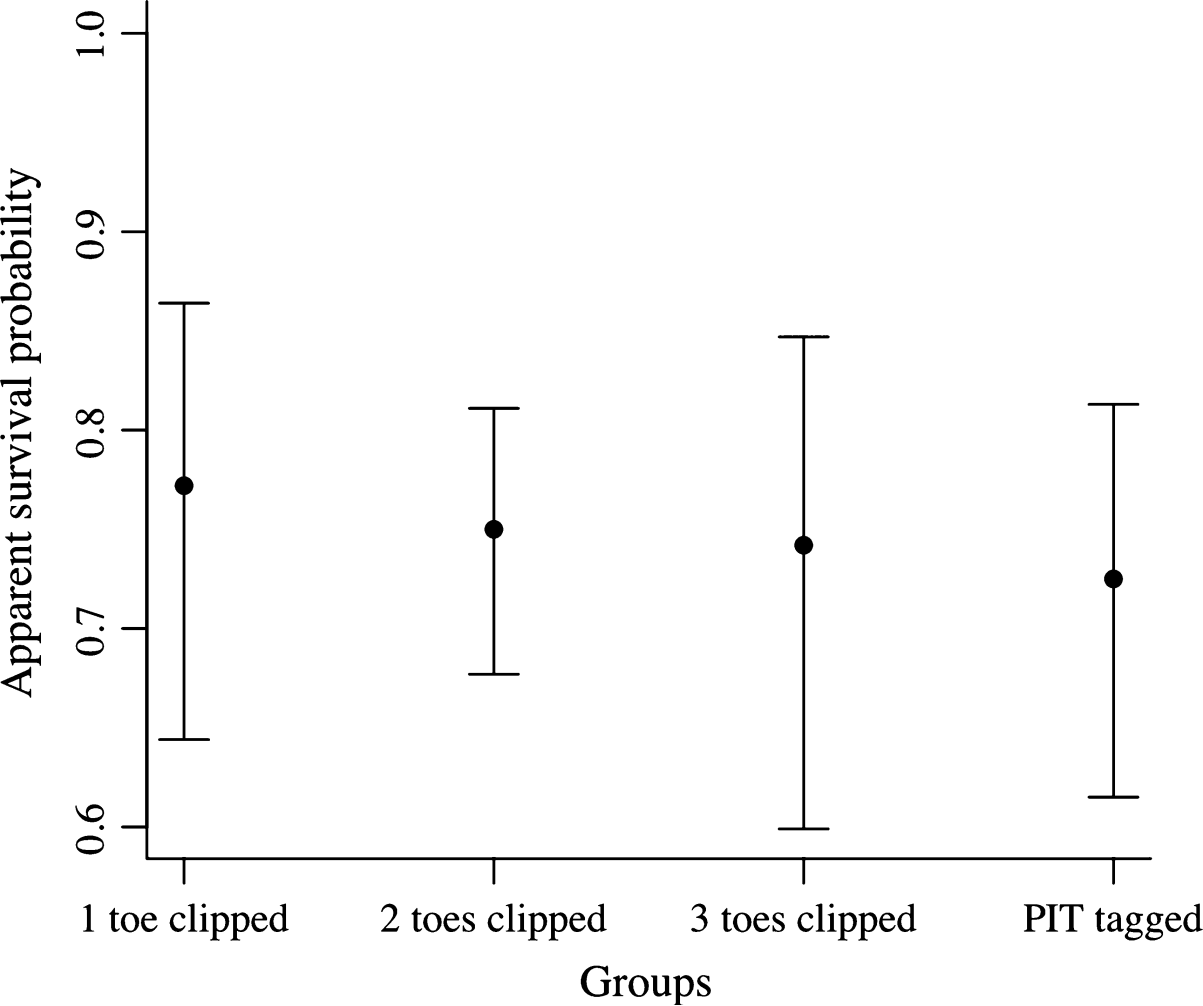
Model-averaged monthly apparent survival probability (and 95% CI) among groups.

The top model (AIC*c* weight = 0.07) included constant apparent survival and detection probability varying as an additive effect of sex and linear trend on toe pad-clipped groups. However, models had similar AIC*c* weights, with considerable model selection uncertainty (Appendix B). Considering all models averaged, apparent monthly survival probability was similar among the four groups, with slightly higher survival probability for individuals with one toe pad clipped (0.77), then two (0.75) and three (0.74) toe pads clipped, similar to McCarthy and Parris (2004). Survival was the lowest for PIT-tagged individuals (0.72), but there was also considerable uncertainty around the estimates (Fig. 1, Appendix C). Assuming group one's survival estimate as the closest of a control group (as we have no estimates for individuals with no toe pads clipped), removing the second toe pad reduced survival probability in 2.6% in relation to group one, and the third toe pad in 3.6%. The use of PIT tag reduced survival in 5.8% in relation to group one's point estimate. No covariate was important for describing the apparent survival probability, because all of them presented AIC*c* cumulative model weights below 0.5 (Appendix C).

**Appendix B:** Model results.

Model	AIC*c*	ΔAIC*c*	*w*	*k*	Deviance
Phi(.) p(sex+toe_lin)	849.53	0.00	0.07	4	841.47
Phi(.) p(sex+tech)	850.27	0.74	0.05	4	842.20
Phi(tech) p(sex)	850.91	1.38	0.04	4	842.85
Phi(sex) p(sex+toe_lin)	850.93	1.41	0.04	5	840.84
Phi(.) p(sex)	850.95	1.42	0.04	3	844.91
Phi(tech) p(sex+toe_lin)	851.29	1.76	0.03	5	841.19
Phi(.) p(sex+tech+toe_lin)	851.54	2.01	0.03	5	841.45
Phi(toe_lin) p(sex+toe_lin)	851.55	2.03	0.03	5	841.46
Phi(toe_lin) p(sex)	851.66	2.13	0.03	4	843.60
Phi(tech+toe_lin) p(sex)	851.68	2.15	0.03	5	841.58
Phi(sex) p(sex+tech)	851.70	2.17	0.03	5	841.60
Phi(tech) p(sex+tech)	851.98	2.46	0.02	5	841.89
Phi(tech+toe_lin) p(sex+toe_lin)	852.13	2.60	0.02	6	840.00
Phi(toe_lin) p(sex+tech)	852.27	2.74	0.02	5	842.18
Phi(sex) p(sex)	852.33	2.80	0.02	4	844.27
Phi(tech+toe_lin) p(sex+toe_lin)	852.44	2.91	0.02	6	840.30
Phi(sex+toe_lin) p(sex)	852.62	3.09	0.02	5	842.53
Phi(tech+toe_lin) p(sex)	852.76	3.23	0.01	5	842.67
Phi(sex) p(sex+tech+toe_lin)	852.95	3.42	0.01	6	840.81
Phi(sex+toe_lin) p(sex+toe_lin)	852.96	3.43	0.01	6	840.83
Phi(sex+tech) p(sex+tech)	853.12	3.59	0.01	6	840.98
Phi(tech) p(sex+tech+toe_lin)	853.22	3.69	0.01	6	841.09
Phi(tech+toe_lin) p(sex+tech+toe_lin)	853.31	3.78	0.01	7	839.13
Phi(.) p(sex)	853.36	3.83	0.01	6	841.22
Phi(.) p(g+sex+tech)	853.36	3.83	0.01	6	841.22
Phi(.) p(g+sex+toe_lin)	853.36	3.83	0.01	6	841.22
Phi(.) p(g+sex+tech+toe_lin)	853.36	3.83	0.01	6	841.22
Phi(sex+tech+toe_lin) p(sex+toe_lin)	853.44	3.91	0.01	7	839.26
Phi(sex+tech+toe_lin) p(sex)	853.53	4.00	0.01	6	841.40
Phi(toe_lin) p(sex+tech+toe_lin)	853.57	4.05	0.01	6	841.44
Phi(sex+toe_lin) p(sex+tech)	853.61	4.08	0.01	6	841.47
Phi(tech+toe_lin) p(sex+tech)	853.65	4.12	0.01	6	841.51
Phi(g) p(sex+toe_lin)	853.75	4.22	0.01	7	839.57
Phi(g+tech) p(sex+toe_lin)	853.75	4.22	0.01	7	839.57
Phi(g+toe_lin) p(sex+toe_lin)	853.75	4.22	0.01	7	839.57
p(g+tech+toe_lin) p(sex+toe_lin)	853.75	4.22	0.01	7	839.57
Phi(g) p(sex)	853.75	4.22	0.01	6	841.62
Phi(g+tech) p(sex)	853.75	4.22	0.01	6	841.62
Phi(g+toe_lin) p(sex)	853.75	4.22	0.01	6	841.62
Phi(g+tech+toe_lin) p(sex)	853.75	4.22	0.01	6	841.62
Phi(sex+tech) p(sex+tech+toe_lin)	854.29	4.76	0.01	7	840.11
Phi(sex+tech+toe_lin) p(sex+tech+toe_lin)	854.46	4.93	0.01	8	838.23
Phi(g+sex) p(sex)	854.53	5.00	0.01	7	840.35
Phi(g+sex+tech) p(sex)	854.53	5.00	0.01	7	840.35
Phi(g+sex+toe_lin) p(sex)	854.53	5.00	0.01	7	840.35
Phi(g+sex+tech+toe_lin) p(sex)	854.53	5.00	0.01	7	840.35
Phi(sex) p(g+sex)	854.75	5.22	0.01	7	840.58
Phi(sex) p(g) p(sex+tech)	854.75	5.22	0.01	7	840.58
Phi(sex) p(g+sex+toe_lin)	854.75	5.22	0.01	7	840.58
Phi(sex) p(g+sex+tech+toe_lin)	854.75	5.22	0.01	7	840.58
Phi(sex+tech+toe_lin) p(sex+tech)	854.83	5.30	0.01	7	840.65
Phi(g) p(sex+tech)	854.92	5.39	0.01	7	840.75
Phi(g+tech) p(sex+tech)	854.92	5.39	0.01	7	840.75
Phi(g+toe_lin) p(sex+tech)	854.92	5.39	0.01	7	840.75
Phi(g+tech+toe_lin) p(sex+tech)	854.92	5.39	0.01	7	840.75
Phi(sex+toe_lin) p(sex+tech+toe_lin)	854.97	5.44	0.00	7	840.80
Phi(g+sex) p(sex+toe_lin)	855.00	5.47	0.00	8	838.77
Phi(g+sex+tech) p(sex+toe_lin)	855.00	5.47	0.00	8	838.77
Phi(g+sex+toe_lin) p(sex+toe_lin)	855.00	5.47	0.00	8	838.77
Phi(tech) p(g+sex)	855.02	5.49	0.00	7	840.84
Phi(tech) p(g+sex+tech)	855.02	5.49	0.00	7	840.84
Phi(tech) p(g+sex+toe_lin)	855.02	5.49	0.00	7	840.84
Phi(tech) p(g+sex+tech+toe_lin)	855.02	5.49	0.00	7	840.84
Phi(g) p(sex+tech+toe_lin)	855.08	5.56	0.00	8	838.86
Phi(g+tech) p(sex+tech+toe_lin)	855.08	5.56	0.00	8	838.86
Phi(g+toe_lin) p(sex+tech+toe_lin)	855.08	5.56	0.00	8	838.86
Phi(tech+toe_lin) p(g+sex)	855.10	5.57	0.00	8	838.87
Phi(tech+toe_lin) p(g+sex+tech)	855.10	5.57	0.00	8	838.87
Phi(tech+toe_lin) p(g+sex+toe_lin)	855.10	5.57	0.00	8	838.87
Phi(toe_lin) p(g+sex)	855.40	5.87	0.00	7	841.22
Phi(toe_lin) p(g+sex+tech)	855.40	5.87	0.00	7	841.22
Phi(toe_lin) p(g+sex+toe_lin)	855.40	5.87	0.00	7	841.22
Phi(toe_lin) p(g+sex+tech+toe_lin)	855.40	5.87	0.00	7	841.22
Phi(g+sex) p(sex+tech)	856.05	6.52	0.00	8	839.82
Phi(g+sex+tech) p(sex+tech)	856.05	6.52	0.00	8	839.82
Phi(g+sex+toe_lin) p(sex+tech)	856.05	6.52	0.00	8	839.82
Phi(sex+tech) p(g+sex)	856.05	6.53	0.00	8	839.83
Phi(sex+tech) p(g+sex+tech)	856.05	6.53	0.00	8	839.83
Phi(sex+tech) p(g+sex+toe_lin)	856.05	6.53	0.00	8	839.83
Phi(g+sex) p(sex+tech+toe_lin)	856.20	6.67	0.00	9	837.92
Phi(g+sex+tech) p(sex+tech+toe_lin)	856.20	6.67	0.00	9	837.92
Phi(g+sex+toe_lin) p(sex+tech+toe_lin)	856.20	6.67	0.00	9	837.92
Phi(g+sex+tech+toe_lin) p(sex+tech+toe_lin)	856.20	6.67	0.00	9	837.92
Phi(sex+tech+toe_lin) p(g+sex)	856.23	6.70	0.00	9	837.94
Phi(sex+tech+toe_lin) p(g+sex+tech)	856.23	6.70	0.00	9	837.94
Phi(sex+tech+toe_lin) p(g+sex+toe_lin)	856.23	6.70	0.00	9	837.94
Phi(sex+tech+toe_lin) p(g+sex+tech+toe_lin)	856.23	6.70	0.00	9	837.94
Phi(sex+toe_lin) p(g+sex)	856.78	7.25	0.00	8	840.55
Phi(sex+toe_lin) p(g+sex+tech)	856.78	7.25	0.00	8	840.55
Phi(sex+toe_lin) p(g+sex+toe_lin)	856.78	7.25	0.00	8	840.55
Phi(sex+toe_lin) p(g+sex+tech+toe_lin)	856.78	7.25	0.00	8	840.55
Phi(g+sex+tech+toe_lin p(sex+toe_lin)	857.06	7.53	0.00	9	838.77
Phi(.) phi g p(g+sex)	857.07	7.54	0.00	9	838.79
Phi(g+tech) p(g+sex)	857.07	7.54	0.00	9	838.79
Phi(g+toe_lin) p(g+sex)	857.07	7.54	0.00	9	838.79
Phi(g) p(g+sex+tech)	857.07	7.54	0.00	9	838.79
Phi(g) p(g+sex+toe_lin)	857.07	7.54	0.00	9	838.79
Phi(g+tech) p(g+sex+toe_lin)	857.07	7.54	0.00	9	838.79
Phi(g+toe_lin) p(g+sex+toe_lin)	857.07	7.54	0.00	9	838.79
Phi(g+tech+toe_lin) p(g+sex+toe_lin)	857.07	7.54	0.00	9	838.79
Phi(g) p(g+sex+tech+toe_lin)	857.07	7.54	0.00	9	838.79
Phi(g+tech) p(g+sex+tech+toe_lin)	857.07	7.54	0.00	9	838.79
Phi(g+tech+toe_lin) p(sex+tech+toe_lin)	857.14	7.61	0.00	9	838.86
Phi(tech+toe_lin) p(g+sex+tech+toe_lin)	857.16	7.63	0.00	9	838.87
Phi(g+sex+tech+toe_lin) p(sex+tech)	858.11	8.58	0.00	9	839.82
Phi(sex+tech) p(g) p(sex+tech+toe_lin)	858.11	8.58	0.00	9	839.83
Phi(g+sex) p(g+sex)	858.19	8.66	0.00	10	837.84
Phi(g+sex+tech) p(g+sex)	858.19	8.66	0.00	10	837.84
Phi(g+sex+toe_lin) p(g+sex)	858.19	8.66	0.00	10	837.84
Phi(g+sex) p(g+sex+tech)	858.19	8.66	0.00	10	837.84
Phi(g+sex+tech) p(g+sex+tech)	858.19	8.66	0.00	10	837.84
Phi(g+sex+toe_lin) p(g+sex+tech)	858.19	8.66	0.00	10	837.84
Phi(g+sex) p(g+sex+toe_lin)	858.19	8.66	0.00	10	837.84
Phi(g+sex+tech) p(g+sex+toe_lin)	858.19	8.66	0.00	10	837.84
Phi(g+tech+toe_lin) p(g+sex)	859.14	9.61	0.00	10	838.79
Phi(g+tech) p(g+sex+tech)	859.14	9.61	0.00	10	838.79
Phi(g+toe_lin) p(g+sex+tech)	859.14	9.61	0.00	10	838.79
Phi(g+tech+toe_lin) p(g+sex+tech)	859.14	9.61	0.00	10	838.79
Phi(g+toe_lin) p(g+sex+tech+toe_lin)	859.14	9.61	0.00	10	838.79
Phi(g+sex+tech+toe_lin) p(g+sex)	860.26	10.73	0.00	11	837.84
Phi(g+sex+toe_lin) p(g+sex+toe_lin)	860.26	10.73	0.00	11	837.84
Phi(g+sex) p(g+sex+tech+toe_lin)	860.26	10.73	0.00	11	837.84
Phi(g+sex+toe_lin) p(g+sex+tech+toe_lin)	860.26	10.73	0.00	11	837.84
Phi(g+tech+toe_lin) p(g+sex+tech+toe_lin)	861.21	11.68	0.00	11	838.79
Phi(g+sex+tech+toe_lin) p(g+sex+tech)	862.34	12.81	0.00	12	837.84
Phi(g+sex+tech+toe_lin) p(g+sex+toe_lin)	862.34	12.81	0.00	12	837.84
Phi(g+sex+tech) p(g+sex+tech+toe_lin)	862.34	12.81	0.00	12	837.84
Phi(g+sex+tech+toe_lin) p(g+sex+tech+toe_lin)	866.51	16.98	0.00	14	837.84
Phi(sex) p(toe_lin)	870.13	20.60	0.00	4	862.07
Phi(sex) p(tech)	870.94	21.41	0.00	4	862.88
Phi(sex+toe_lin) p(toe_lin)	871.07	21.54	0.00	5	860.98
Phi(sex+tech+toe_lin) p(toe_lin)	871.98	22.45	0.00	6	859.85
Phi(sex) p(.)	871.99	22.46	0.00	3	865.95
Phi(sex+tech) p(toe_lin)	872.05	22.52	0.00	5	861.96
Phi(sex) p(tech+toe_lin)	872.14	22.62	0.00	5	862.05
Phi(sex+toe_lin) p(tech)	872.43	22.90	0.00	5	862.33
Phi(sex+tech) p(tech)	872.85	23.32	0.00	5	862.75
Phi(sex+toe_lin) p(tech+toe_lin)	873.10	23.57	0.00	6	860.97
Phi(sex+tech) p(.)	873.35	23.82	0.00	4	865.29
Phi(sex+tech+toe_lin) p(tech+toe_lin)	873.35	23.82	0.00	7	859.17
Phi(g+sex) p(toe_lin)	873.60	24.07	0.00	7	859.42
Phi(g+sex+tech) p(toe_lin)	873.60	24.07	0.00	7	859.42
Phi(g+sex+toe_lin) p(toe_lin)	873.60	24.07	0.00	7	859.42
Phi(sex+toe_lin) p(.)	873.75	24.22	0.00	4	865.68
Phi(sex) p(g)	873.83	24.30	0.00	6	861.69
Phi(sex) p(g+tech)	873.83	24.30	0.00	6	861.69
Phi(sex) p(g+toe_lin)	873.83	24.30	0.00	6	861.69
Phi(sex+tech) p(tech+toe_lin)	874.09	24.56	0.00	6	861.95
Phi(sex+tech+toe_lin) p(tech)	874.22	24.69	0.00	6	862.08
Phi(sex+toe_lin) p(g)	874.85	25.32	0.00	7	860.67
Phi(sex+toe_lin) p(g+tech)	874.85	25.32	0.00	7	860.67
Phi(sex+toe_lin) p(g+toe_lin)	874.85	25.32	0.00	7	860.67
Phi(sex+tech+toe_lin) p(g)	875.03	25.50	0.00	8	858.80
Phi(sex+tech+toe_lin) p(g+tech)	875.03	25.50	0.00	8	858.80
Phi(sex+tech+toe_lin) p(g+toe_lin)	875.03	25.50	0.00	8	858.80
Phi(g+sex) p(tech+toe_lin)	875.09	25.56	0.00	8	858.86
Phi(g+sex+tech) p(tech+toe_lin)	875.09	25.56	0.00	8	858.86
Phi(g+sex+toe_lin) p(tech+toe_lin)	875.09	25.56	0.00	8	858.86
Phi(sex+tech+toe_lin p(.)	875.09	25.57	0.00	5	865.00
Phi(g+sex) p(tech)	875.49	25.96	0.00	7	861.32
Phi(g+sex+tech) p(tech)	875.49	25.96	0.00	7	861.32
Phi(g+sex+toe_lin) p(tech)	875.49	25.96	0.00	7	861.32
Phi(g+sex+tech+toe_lin) p(tech)	875.49	25.96	0.00	7	861.32
Phi(g+sex+tech+toe_lin) p(toe_lin)	875.65	26.12	0.00	8	859.42
Phi(sex+tech) p(g)	875.80	26.27	0.00	7	861.62
Phi(sex+tech) p(g+tech)	875.80	26.27	0.00	7	861.62
Phi(sex+tech) p(g+toe_lin)	875.80	26.27	0.00	7	861.62
Phi(sex+tech) p(g+tech+toe_lin)	875.80	26.27	0.00	7	861.62
Phi(sex) p(g+tech+toe_lin)	875.87	26.34	0.00	7	861.69
Phi(g+sex) p(.)	876.02	26.49	0.00	6	863.89
Phi(g+sex+tech) p(.)	876.02	26.49	0.00	6	863.89
Phi(g+sex+toe_lin) p(.)	876.02	26.49	0.00	6	863.89
Phi(g+sex+tech+toe_lin) p(.)	876.02	26.49	0.00	6	863.89
Phi(sex+toe_lin) p(g+tech+toe_lin)	876.90	27.37	0.00	8	860.67
Phi(g+sex) p(g)	877.02	27.49	0.00	9	858.73
Phi(g+sex+tech) p(g)	877.02	27.49	0.00	9	858.73
Phi(g+sex+toe_lin) p(g)	877.02	27.49	0.00	9	858.73
Phi(g+sex) p(g+tech)	877.02	27.49	0.00	9	858.73
Phi(g+sex+tech) p(g+tech)	877.02	27.49	0.00	9	858.73
Phi(g+sex+toe_lin) p(g+tech)	877.02	27.49	0.00	9	858.73
Phi(g+sex) p(g+toe_lin)	877.02	27.49	0.00	9	858.73
Phi(g+sex+toe_lin) p(g+toe_lin)	877.02	27.49	0.00	9	858.73
Phi(g+sex+tech+toe_lin) p(g+toe_lin)	877.02	27.49	0.00	9	858.73
Phi(sex+tech+toe_lin) p(g+tech+toe_lin)	877.08	27.55	0.00	9	858.80
Phi(g+sex+tech+toe_lin) p(tech+toe_lin)	877.14	27.62	0.00	9	858.86
Phi(g+sex+tech+toe_lin) p(g)	879.08	29.55	0.00	10	858.73
Phi(g+sex+tech+toe_lin) p(g+tech)	879.08	29.55	0.00	10	858.73
Phi(g+sex+tech) p(g+toe_lin)	879.08	29.55	0.00	10	858.73
Phi(g+sex) p(g+tech+toe_lin)	879.08	29.55	0.00	10	858.73
Phi(g+sex+toe_lin) p(g+tech+toe_lin)	879.08	29.55	0.00	10	858.73
Phi(g+sex+tech+toe_lin) p(g+tech+toe_lin)	879.08	29.55	0.00	10	858.73
Phi(.) p(toe_lin)	880.41	30.88	0.00	3	874.37
Phi(g+sex+tech) p(g+tech+toe_lin)	881.15	31.62	0.00	11	858.73
Phi(.) p(tech)	881.33	31.80	0.00	3	875.29
Phi(.) p(.)	881.96	32.43	0.00	2	877.94
Phi(tech) p(toe_lin)	882.33	32.80	0.00	4	874.27
Phi(tech) p(.)	882.36	32.83	0.00	3	876.32
Phi(.) p(tech+toe_lin)	882.36	32.83	0.00	4	874.30
Phi(toe_lin) p(toe_lin)	882.36	32.83	0.00	4	874.30
Phi(toe_lin) p(.)	882.95	33.42	0.00	3	876.91
Phi(tech+toe_lin) p(toe_lin)	883.13	33.60	0.00	5	873.04
Phi(tech) p(tech)	883.21	33.69	0.00	4	875.15
Phi(toe_lin) p(tech)	883.35	33.82	0.00	4	875.29
Phi(.) p(g)	883.77	34.24	0.00	5	873.68
Phi(.) p(g+tech)	883.77	34.24	0.00	5	873.68
Phi(.) p(g+toe_lin)	883.77	34.24	0.00	5	873.68
Phi(.) p(g+tech+toe_lin)	883.77	34.24	0.00	5	873.68
Phi(tech+toe_lin) p(tech) p(toe_lin)	884.09	34.56	0.00	6	871.96
Phi(tech) p(tech+toe_lin)	884.21	34.69	0.00	5	874.12
Phi(tech+toe_lin) p(.)	884.24	34.71	0.00	4	876.18
Phi(toe_lin) p(tech+toe_lin)	884.33	34.80	0.00	5	874.24
Phi(g) p(toe_lin)	884.47	34.94	0.00	6	872.34
Phi(g+tech) p(toe_lin)	884.47	34.94	0.00	6	872.34
Phi(g+toe_lin) p(toe_lin)	884.47	34.94	0.00	6	872.34
Phi(g+tech+toe_lin) p(toe_lin)	884.47	34.94	0.00	6	872.34
Phi(g) p(.)	884.72	35.19	0.00	5	874.63
Phi(g+tech) p(.)	884.72	35.19	0.00	5	874.63
Phi(g+toe_lin) p(.)	884.72	35.19	0.00	5	874.63
Phi(tech+toe_lin) p(tech)	884.90	35.38	0.00	5	874.81
Phi(tech+toe_lin) p(g)	885.48	35.95	0.00	7	871.30
Phi(tech+toe_lin) p(g+tech)	885.48	35.95	0.00	7	871.30
Phi(tech+toe_lin) p(g+toe_lin)	885.48	35.95	0.00	7	871.30
Phi(tech) p(g)	885.60	36.07	0.00	6	873.46
Phi(tech) p(g+tech)	885.60	36.07	0.00	6	873.46
Phi(tech) p(g+toe_lin)	885.60	36.07	0.00	6	873.46
Phi(g) p(tech+toe_lin)	885.68	36.15	0.00	7	871.50
Phi(g+tech) p(tech) p(toe_lin)	885.68	36.15	0.00	7	871.50
Phi(g+toe_lin) p(tech+toe_lin)	885.68	36.15	0.00	7	871.50
Phi(toe_lin) p(g)	885.77	36.24	0.00	6	873.64
Phi(toe_lin) p(g+tech)	885.77	36.24	0.00	6	873.64
Phi(toe_lin) p(g+toe_lin)	885.77	36.24	0.00	6	873.64
Phi(toe_lin) p(g+tech+toe_lin)	885.77	36.24	0.00	6	873.64
Phi(g) p(tech)	885.77	36.24	0.00	6	873.64
Phi(g+tech) p(tech)	885.77	36.24	0.00	6	873.64
Phi(g+toe_lin) p(tech)	885.77	36.24	0.00	6	873.64
Phi(g+tech+toe_lin) p(tech)	885.77	36.24	0.00	6	873.64
p(g+tech+toe_lin) p(.)	886.76	37.23	0.00	6	874.63
Phi(g) p(g)	887.46	37.93	0.00	8	871.23
Phi(g+tech) p(g)	887.46	37.93	0.00	8	871.23
Phi(g+toe_lin) p(g)	887.46	37.93	0.00	8	871.23
Phi(g) p(g+tech)	887.46	37.93	0.00	8	871.23
Phi(g+toe_lin) p(g+tech)	887.46	37.93	0.00	8	871.23
Phi(g+tech+toe_lin) p(g+tech)	887.46	37.93	0.00	8	871.23
Phi(g) p(g+toe_lin)	887.46	37.93	0.00	8	871.23
Phi(g+tech) p(g+toe_lin)	887.46	37.93	0.00	8	871.23
Phi(tech+toe_lin) p(g+tech+toe_lin)	887.53	38.00	0.00	8	871.30
Phi(tech) p(g+tech+toe_lin)	887.64	38.11	0.00	7	873.46
Phi(g+tech+toe_lin) p(g)	889.52	39.99	0.00	9	871.23
Phi(g+tech) p(g+tech)	889.52	39.99	0.00	9	871.23
Phi(g+toe_lin) p(g+toe_lin)	889.52	39.99	0.00	9	871.23
Phi(g) p(g+tech+toe_lin)	889.52	39.99	0.00	9	871.23
Phi(g+toe_lin) p(g+tech+toe_lin)	889.52	39.99	0.00	9	871.23
p(g+tech+toe_lin) p(g+tech+toe_lin)	889.52	39.99	0.00	9	871.23
p(g+tech+toe_lin) p(tech+toe_lin)	889.79	40.26	0.00	9	871.50
p(g+tech+toe_lin) p(g+toe_lin)	891.58	42.05	0.00	10	871.23
Phi(g+tech) p(g+tech+toe_lin)	891.58	42.05	0.00	10	871.23

AIC*c*, Akaike's information criteria with small sample size correction; ΔAIC*c*, difference between top model and the current model; *w*_*i*_, AIC*c* weights; *K*, number of parameters; Deviance, difference of the current model and the saturated model. Parameter abbreviations: (.), constant; (sex), varies by sex; (g), varies by group; (toe_lin), varies with linear trend effect; (tech), varies by marking technique.

**Appendix C:** Cumulative AIC*c* weights for the covariates used for apparent survival (Φ) and recapture probability (*P*).

Variables	Φ	*P*
sex	0.35	0.98
group	0.20	0.18
tech	0.40	0.38
toe_lin	0.35	0.48

**Figure 2:**
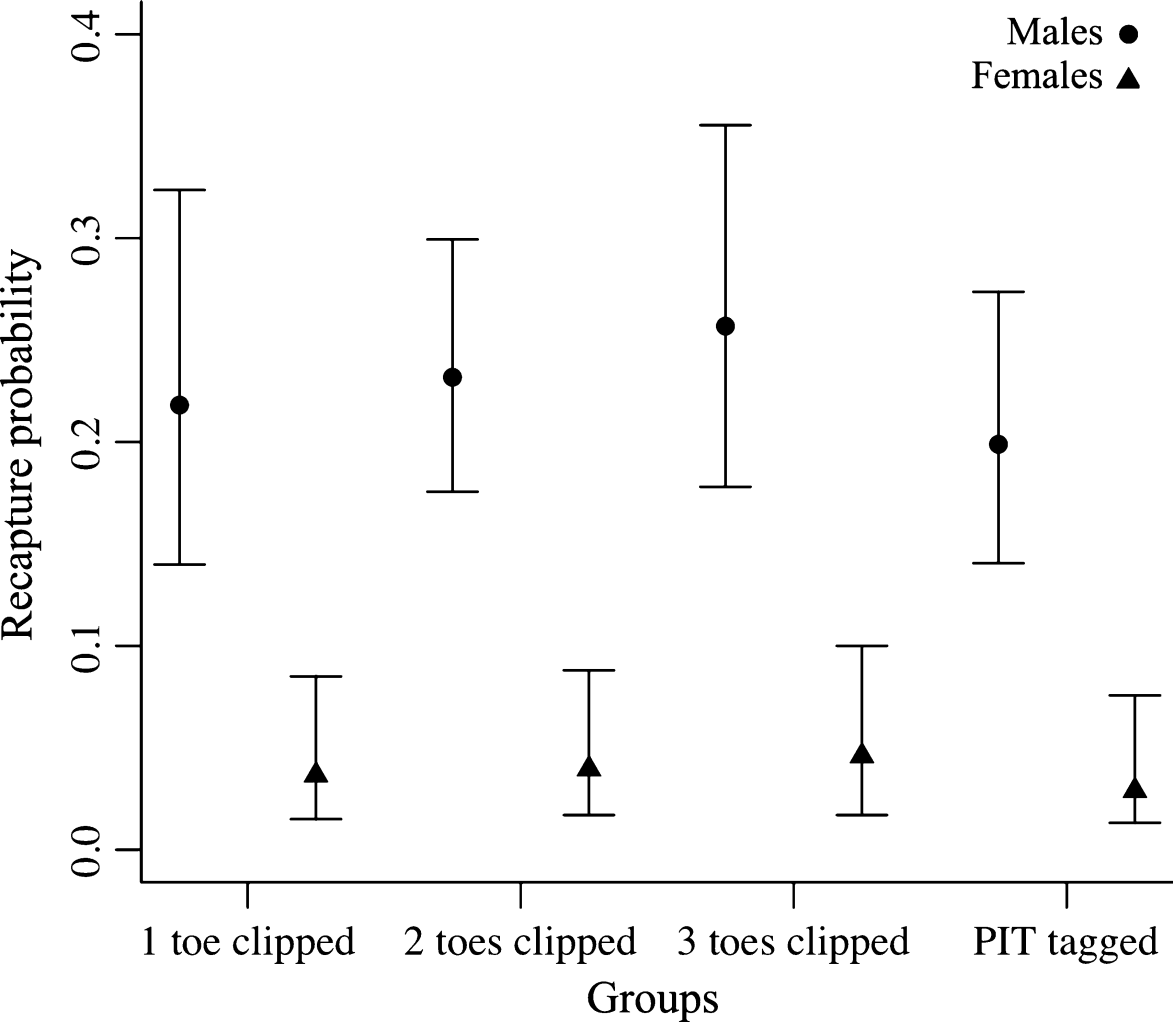
Model-averaged monthly recapture probability (and 95% CI) among groups and sexes.

PIT tagged males and females presented lower recapture probability than toe pad-clipped groups, but confidence intervals greatly overlapped (Fig. 2). The same trend was also showed by the *β* estimate (*β*_technique_ = 0.35, CI −0.07 to 0.8, second top model). The AIC*c* model weight of the linear trend effect on toe pad clipping was close to the 0.5 cutoff (Appendix B), with a slightly higher probability of recapture with the increase of toe pads removed, but confidence intervals overlapped among groups and included zero (*β*_toe_lin_ = 0.15, CI −0.01 to 0.3, top model). Assuming individuals with one toe pad clipped as the closest of a control group, we observed an increase on recapture probability of 6% and 8% for individuals with two toe pads clipped, 17% and 26% for individuals with three toe pads clipped, and a decrease of 9% and 21% for PIT-tagged individuals in males and females, respectively. Sex was the most important covariate predicting recapture probability (0.98 of model weight; Appendix C), where males (from 0.20 to 0.26) and females (from 0.03 to 0.05) greatly differed (Fig. 2).

## Discussion

We found subtle differences in survival probability among individuals marked with different techniques. Although there was uncertainty around the estimates, we should look at the potential biological differences among groups. Looking only at the point estimates, we see that survival probability of individuals with only one toe pad clipped was slightly higher than other groups, declining 2.6% with two toe pads clipped, 3.6% with three toe pads clipped, and finally declining 5.8% in PIT-tagged individuals. Our estimated declines were lower than those predicted by McCarthy and Parris (2004) that reported declines from 4% to 11% when clipping two and three toes respectively. The difference between both studies may relate to different species and procedures used, but also because they used return rates that confound survival and recapture probabilities. Overall, clipping multiple toes can reduce survival substantially.

Differences were observed among groups concerning recapture probability, but confidence intervals overlapped. Among toe pad-clipped groups, recapture probability increased with more toe pads clipped – from 6% to 26% for males and females in individuals with two and three toe pads removed. This may be related to reduction of individual mobility due to the removal of toe pads. Additionally, recapture probability of individuals with two and three toe pads removed could be higher because more individuals were allocated to those groups, or even because individuals in group one were marked at the onset of the first reproductive season, and they could leave the site first. All these hypotheses would be better addressed randomly assigning individuals to the different groups since the beginning of the study. PIT-tagged individuals presented the lowest recapture probability, decreasing 9% and 21% in males and females, respectively. Sex was the strongest covariate influencing recapture probability, carrying the most cumulative AIC*c* weight.

Toe clipping may be a stressor for amphibians if compared to handling only (Narayan et al. 2011; but see Fisher et al. 2013), and negative effects of toe clipping on frog survival and capturing have been observed previously (Lemckert 1996; van Gelder and Strijbosch 1996; Hartel and Nemes 2006). In our study, the removal of toe pads was quicker than marking with the PIT tag, and bleeding usually did not occur. The application of the PIT tag took from two to four times longer (pers. obs. M.G.), and possibly increased handling stress. Many males, especially from toe pad-clipping groups, were seen in normal reproductive activities right after being manipulated and no injures possibly caused by toe clipping were observed during the study. Despite its low cost and ease of use, the number of individuals to be marked using toe clipping/tipping is limited and should always be the smallest as possible. In our study, we did not find evidence of toe regeneration, but if it occurs, as described by Hoffmann et al. (2008), the mark–recapture assumption of mark retention will be violated, underestimating survival and other vital rates (Lebreton et al. 1992; Williams et al. 2002).

Males and females *H*. *faber* present behavioral differences (Duellman and Trueb 1986; Martins and Haddad 1988; Martins 1993). Females spend less time in the ponds, and this may explain the lower recapture probability observed for them. The difference in the recapture probability of males and females indicates that pooling the sexes in the analysis would mask results. In this way, considering the return rate a survival estimate would have provided underestimated estimates (Martin et al. 1995). The importance of sex to our analysis highlights the importance of considering individual (and also temporal, though not used here) covariates when studying potential effects of marking techniques. Most of the articles so far (Waddle et al. 2008; Grafe et al. 2011) do not present comparisons including such covariates.

The use of PIT tags in anurans might be less common than toe clipping, but may be a reliable technique for certain species and has not generally been demonstrated to cause serious problems, such as detrimental effects on body condition or mortality (Christy 1996; Brown 1997; Jehle and Hödl 1998; McAllister et al. 2004; but see Scherer et al. 2005). However, PIT tagging is more costly, increases handling time, requires more skill from the field biologist, and may be unfeasible in small frogs. We are unable to clearly demonstrate the difference between both marking techniques based only on our data and the effects and differences among techniques should be emphasized in future studies. Assuming the difference is real, the stress of higher handling time and PIT tag implant procedure could make individuals to leave the reproductive site, reducing their recapture probability. In this case, individuals would need more time to recover, after being tagged. In addition, while being considered a permanent marking technique (Gibbons and Andrews 2004), PIT tags could be expelled from (Roark and Dorcas 2000) or migrate to another location in the body (Tracy et al. 2011) causing apparent tag loss and affecting population estimates.

In general, studies looking at the impacts of different marking techniques on vital rates of wild populations are scarce. Studies comparing toe clipping and PIT tagging have shown similar effects on survival and growth rates of salamanders (Ott and Scott 1999) and free-living naked mole rats (Braude and Ciszek 1998). It is also noteworthy that the effects of marking techniques vary by species, reproductive strategies, habitats (e.g., arboreal vs. fossorial), and behaviors (Liner and Smith 2007). Frog species will respond in different ways to marking and investigators must consider the characteristics of each species, as well as the use of the most practical and least harmful technique, evaluating all methods together, as suggested by Phillott et al. (2008). Stress response should be included as an important trait to be measured in individuals (Perry et al. 2011), but few studies considered this trait when testing the impacts of different invasive marking techniques on amphibians.

Estimating vital rates of a control group of nonmarked individuals in the field would be ideal, as we were unable to compare survival of individuals that were not marked, and because we believe that both techniques may decrease survival. Perry et al. (2011) suggest the use of Visual Implanted Elastomers (VIE) as a true control group, but handling and inserting the elastomers under a frog's skin might also cause stress. Photography may be a good candidate for a control group in free-ranging populations. Photography also presents problems, like identifiable characteristics on the target species and obtaining good quality pictures without disturbing individuals, as handling only may be an important stressor itself (Fisher et al. 2013). Controlled laboratory experiments may be useful for the inclusion of a nonmarked group, for comparing survival (e.g., looking for inflammation or stress responses) and for allowing estimation of tag loss (Brown 1997). However, laboratory experiments are not the best solution to observe the effects of marking on species interactions (e.g., predation, competition) as well as the effects of weather variability on marked individuals.

In summary, we showed slight differences between both marking techniques. Considering only the statistical results would make us conclude that both techniques performed similarly. Although not discussed here, the decision to adopt a particular marking method should be multidisciplinary, also involving law, ethics and philosophy. However, given the urge of studying and preserving populations, as well as the pros and cons of each technique, a decision has to be put into practice. No perfect technique is available, but being aware of the problems and accounting for the effect of the chosen technique in the analysis is better than ignoring such problems. Based on our estimates of survival and recapture, but also given the lack of comparisons among alternative marking techniques in the literature, as well as logistical issues, such as budget and processing time, we agree with others authors and recommend the use of toe clipping instead of PIT tagging with the blacksmith tree frog. However, there should be a threshold, where toe clipping is not worthwhile (when removing multiple toes, for instance) and another technique to individualize frogs should be considered.
